# Matrix-assisted laser desorption/ionization time of flight mass spectrometry identification of *Vibrio* (*Listonella*) *anguillarum* isolated from sea bass and sea bream

**DOI:** 10.1371/journal.pone.0225343

**Published:** 2019-11-18

**Authors:** Snježana P. Kazazić, Natalija Topić Popović, Ivančica Strunjak-Perović, Sanja Babić, Daniela Florio, Marialetizia Fioravanti, Krunoslav Bojanić, Rozelindra Čož-Rakovac

**Affiliations:** 1 Laboratory for Mass Spectrometry and Functional Proteomics, Rudjer Bošković Institute, Zagreb, Croatia; 2 Laboratory for Aquaculture Biotechnology, Rudjer Bošković Institute, Zagreb, Croatia; 3 Centre of Excellence for Marine Bioprospecting-BioProCro, Rudjer Bošković Institute, Zagreb, Croatia; 4 Department of Veterinary Medical Sciences, Alma Mater Studiorum Università di Bologna, Ozzano Emilia, Italy; Fisheries and Oceans Canada, CANADA

## Abstract

*Vibrio* (*Listonella*) *anguillarum* is a pathogenic bacterium causing septicaemia in a wide range of marine organisms and inducing severe mortalities, thus it is crucial to conduct its accurate and rapid identification. The aim of this study was to assess MALDI-TOF MS as a method of choice for identification of clinical *V*. *anguillarum* isolates from affected marine fish. Since the method accuracy might be influenced by the type of the medium used, as well as by the incubation conditions, we tested *V*. *anguillarum* isolates grown on standard media with and without the addition of NaCl, cultured at three incubation temperatures, and at three incubation periods. The best scores were retrieved for *V*. *anguillarum* strains grown on NaCl-supplemented tryptone soy agar (TSA) at 22°C and incubated for 48h (100% identification to species level; overall score 2.232), followed by incubation at 37°C and 48h (100% to species level; score 2.192). The strains grown on non-supplemented TSA gave the best readings when incubated at 22°C for 72h (100% identification to species level; overall score 2.182), followed by incubation at 15°C for 72h (100% to species level; score 2.160). Unreliable identifications and no-identifications were growing with the incubation duration at 37°C, on both media, amounting to 88.89% for 7d incubation on supplemented TSA, and 92.60% for 7d incubation on non-supplemented TSA. The age of the cultured strains and use of media significantly impacted the mass spectra, demonstrating that for reliable identification, MALDI-TOF MS protein fingerprinting with the on-target extraction should be performed on strains grown on a NaCl-supplemented medium at temperatures between 15 and 22°C, incubated for 48–72 hours.

## Introduction

*Vibrio* (*Listonella*) *anguillarum* is a pathogenic bacterium causing septicaemia (vibriosis) in a wide range of marine organisms, namely fish, crustaceans and bivalves. It induces aquaculture mortalities up to 100%, as it aggressively penetrates a host using a number of genetic virulence factors, and leads to lethal outcomes as soon as three days following initial exposure [1]. *V*. *anguillarum* isolates vary by cellular sugar compositions [2], and consist of at least 23 O-serogroups [3], of which serotypes O1-O5 are reported as virulent [1]. Its recovery by use of culture-based methods is possible from various media, such as tryptone soy agar (TSA), nutrient agar, brain heart infusion agar, thiosulphate citrate bile salts sucrose agar, and particularly VAM medium, but neither is selectively specific for *V*. *anguillarum* exclusively [4–5].

A scheme comprising a combination of diagnostic and clinical methods was introduced by Topić Popović and co-workers [6] for the use of API 20E (Biomerieux, Marcy-l’Etoile, France) protocol, since biochemical tests, although time consuming, were proven rather reliable for its identification [7]. Tools which are not based on metabolic fingerprinting rely on the antigen content of the *V*. *anguillarum* cells, PCR and real-time PCR protocols, dot-blot DNA hybridization (raw DNA and PCR-amplified DNA) by oligonucleotide probes, immunoassays based on ELISA tests, and isothermal methods such as loop-mediated isothermal amplification [8–9]. However, except from water samples, identification of uncultured fish tissue bacteria is a challenge [8]. Matrix-assisted laser desorption/ionization time of flight mass spectrometry (MALDI-TOF MS) was developed to detect ribosomal proteins of cultured bacteria within minutes, with low costs, and high levels of sensitivity, specificity, and throughput. Most of the above mentioned tools and techniques are time-consuming and laborious, requiring highly trained operators, while MALDI-TOF MS, depending on the database, yields reliable identification results in several straight-forward steps [10–11]. The Bruker’s database version DB-5989 currently includes 7 entries of *V*. *anguillarum* (02 EGS, 03 EGS, DSM 11323 DSM, DSM 21597T DSM, LMG 4437T HAM, serotype 02 EGS, serotype 03 EGS).

The aim of this study was to assess MALDI-TOF MS as a method for accurate identification of clinical *V*. *anguillarum* isolates from European sea bass (*Dicentrarchus labrax*) and gilthead sea bream (*Sparus aurata*), previously confirmed by latex agglutination and API 20E system. Since the method accuracy might be influenced by the type of the medium used, as well as by the incubation conditions, we tested *V*. *anguillarum* isolates grown on standard media with and without the addition of NaCl, cultured at three incubation temperatures, and at three incubation periods.

## Material and methods

All applicable international, national and/or institutional guidelines for the care and use of animals were followed and the procedures performed in the study were in accordance with the ethical standards of the institution. The isolation of bacterial strains was performed from brain and kidney of dissected fish during diagnostic activities carried out on fish found dead during mortality outbreaks in farms and sent to the Fish Pathology Laboratory of the Department of Veterinary Medical Sciences of Bologna University to determine the cause of death (Nota Ministero della Salute 0017710-P-26/07/2017—Decreto Legislativo n. 26, 4 marzo 2014).

### Bacterial strains

The *V*. *anguillarum* isolates (confirmed by latex agglutination tests with Mono-Va kit (Bionor, Skien, Norway) and API 20E system (Biomerieux), code 3047524–3047526), were retrieved from the affected marine fish. Fish were European sea bass (*Dicentrarchus labrax*) and gilthead sea bream (*Sparus aurata*) farmed in sea cages and land-based tanks in Italy and Croatia. Fish size varied from fry to adults of 550 g of weight. Strains were frozen at -86°C.

A total of 27 *V*. *anguillarum* strains were used, and cultured on tryptone soy agar (TSA) (Oxoid Ltd, England UK). The medium formula contained pancreatic digest of casein (15g/L), enzymatic digest of soya bean (5 g/L), agar (15g/L), and sodium chloride (5g/L). The non-supplemented medium thus inherently contained 0.5% NaCl. Strains were also cultured on TSA medium supplemented with 1.5% NaCl.

### Matrix-assisted laser desorption/ionization time of flight mass spectrometry identification

Isolates (three biological replicates) were applied on a 96-spot polished stainless steel target plate by the on-target extraction method after being cultured at 15°C, 22°C, and 37°C. The first time-point for identification was 48h, relating to the occurrence of the first colonies on plates containing both growth media. Colonies were subsequently applied on a target plate at the second time-point of 72h and at the final time-point of 7d of growth. They were all analysed by the on-target extraction method. Procedures were conducted in duplicates up to quintuplicates, with a total of 3243 measurements.

The on-target extraction was initiated with a smear of a colony loopful from each strain on a designated spot on the target plate (Bruker Daltonics, Bremen, Germany). Subsequently, 1 μL of 70% formic acid (Kemika, Croatia) was added to each bacterial colony. After drying at room temperature, 1 μL of MALDI matrix was added to each spot, allowed to air dry at 22°C, after which 2 μL of MALDI matrix was added to each spot with a bacterial colony smear (saturated solution of α-cyano-4-hydroxycinnamic acid in 50% acetonitrile and 2.5% trifluoroacetic acid (Bruker Daltonics)).

The measurements were carried out using a bench-top Bruker Microflex LT mass spectrometer equipped with the Bruker Biotyper 3.0 software (Bruker Daltonics), according to the manufacturer’s instructions. For validation of runs, the system was calibrated with a bacterial test standard *Escherichia coli* DH5 alpha spiked with two additional pure proteins (RNAse A and myoglobin) to cover an overall mass range from 4 to 17 kDa. Briefly, ions were captured in the positive linear mode (mass range 2–20 kDa), and positive ions were extracted at accelerated voltage of 20 kV. Spectra with the sum of the respective ions were obtained by 240 laser shots in different regions of every target plate spot.

### MALDI-TOF MS data interpretation

Every mass spectrum was compared to the reference mass spectra in the database. The Biotyper software calculated obtained algorithms and data were recorded as logarithmic scores between 0 and 3.0. As per the manufacturer, a log score of <1.700 was considered unreliable, a score of 1.700 to 1.999 indicated probable identification to the genus level, a score of 2.000 to 2.299 indicated probable species identification, while a log score of 2.300 to 3.000 indicated highly probable species level identification. Specimens failing to yield log scores of 2.000 and above were manually analysed by using targeted laser shots instead of randomly generated by the software. The database used included reference entries with 7 *V*. *anguillarum* species, and presence or absence of peaks of every fingerprinted isolate were matched to these entries.

### Analysis of mass spectra

Spectra were inspected using the MALDI Biotyper Preprocessing Standard Method and the MALDI Biotyper MSP Identification Standard Method adjusted by the manufacturer (Bruker Daltonics, Germany). Dendrogram was created by MALDI Biotyper 3.0 with following settings: distance measure was set at correlation and linkage at average. Distance values were relative and normalized to a maximum value of 1000. Dendrogram was calculated on the basis of cluster analysis of mass spectra of *V*. *anguillarum* isolates.

### Statistical analysis

Logarithmic scores > = 2.000 were categorised as positive and < 2.000 as negative species identifications based on a requirement of attaining probable species identifications to reach a clinical diagnosis for potential subsequent therapeutic interventions. Identification as a dependent variable was evaluated using generalized linear logistic regression models for association with media, temperature, and time of measurement as independent variables. Due to repeated measurements on strains, mixed effects logistic regression was also used with strains as random effects and were compared with fixed effects only regression models. Models were built using a forward step procedure and compared using ANOVA tables. Final model was evaluated using K-fold cross validation with a training set of 75% and a testing set of the remaining 25% of the data using 1,000 replicates. Receiver operating characteristics curves were also used to evaluate performance measures of the model. All statistical tests used the α error set at 0.05 (5% false positive rate). All exploratory and statistical analyses were performed using R software v.3.6.1 (R Core Team (2019). R: A language and environment for statistical computing. R Foundation for Statistical Computing, Vienna, Austria. URL https://www.R-project.org/).

## Results

Overall, MALDI-TOF MS identified 15.22% of isolates accurately only to the genus level and 73.85% to the species level, when observing results irrespective of the NaCl supplementation of the medium and culture conditions. However, when comparing species identification for different culture conditions, the results varied significantly by media type (p<0.001), incubating temperature (p<0.001), and time of measurement (p<0.001).

Identification of strains grown on NaCl-supplemented TSA outperformed identification on non-supplemented TSA (Tables 1 and 2). The best scores were retrieved for *V*. *anguillarum* strains grown on NaCl-supplemented TSA at 22°C and incubated for 48h, followed by incubation at 37°C at 48h. The samples grown on non-supplemented TSA gave the best readings when incubated at 22°C for 72h, followed by incubation at 15°C for 72h. The results obtained for isolates grown at 37°C for 7 days on both media have to be interpreted with caution since they included a significant percent of unreliable scores.

**Table 1:** Identification results (as log scores) regarding *V. anguillarum* incubation time and temperature for strains cultured on Tryptone soy agar (TSA) containing 0.5% NaCl and supplemented with 1.5% NaCl.

Strain ID	15°C			22°C			37°C		
	48h	72h	7d	48h	72h	7d	48h	72h	7d
1 305/C/02	2.102	2.182	2.100	2.167	2.075	1.868	2.213	2.133	1.392
2 68/05	2.136	2.187	1.925	2.047	2.187	1.821	2.077	2.006	1.349
3 82/05	2.062	2.257	1.996	2.203	2.180	1.879	2.149	2.161	1.506
4 372/05	2.118	2.243	2.064	2.138	2.234	1.852	2.237	2.013	1.456
5 31/06	2.058	2.144	2.079	2.201	2.228	1.875	2.195	2.019	1.614
6 136/C/06	2.206	2.265	2.034	2.219	2.256	1.882	2.283	2.228	1.373
7 77/09	2.192	2.208	1.959	2.198	2.226	1.912	2.205	2.145	1.262
8 135/09	2.157	2.220	2.010	2.143	2.219	2.013	2.228	2.140	1.379
9 158/B/09	2.153	2.248	2.227	2.251	2.135	1.934	2.162	2.148	1.313
10 84/10	2.183	**2.371**	2.092	2.290	2.218	1.881	2.149	2.234	1.765
11 246/D/10	2.175	1.724	2.064	2.203	2.210	2.047	2.212	2.131	1.715
12 65/A/11	2.145	2.183	2.148	2.230	2.214	2.006	2.207	2.199	1.819
13 83/A/11	2.141	2.181	2.080	2.196	2.137	1.999	2.236	2.237	1.461
14 104/B/12	2.152	2.291	2.003	2.236	2.148	1.999	2.182	2.206	1.446
15 117/13	2.272	2.271	2.034	2.238	2.165	1.992	**2.316**	2.146	1.450
16 189/13	2.128	2.229	2.041	**2.330**	2.136	1.904	2.210	2.167	1.422
17 440/14	2.243	2.194	2.027	2.249	2.168	1.860	2.124	2.196	1.364
18 449/A/14	2.199	2.254	2.101	2.288	2.073	1.972	2.185	2.185	1.288
19 4/B/15	2.121	2.247	2.069	2.289	2.124	1.951	2.266	2.162	1.384
20 73/15	2.142	2.224	2.054	**2.339**	2.168	2.006	2.132	2.152	1.240
21 121/15	2.152	2.240	2.036	2.299	2.201	1.962	2.213	2.210	1.205
22 130/F/15	2.193	2.134	2.045	2.275	2.090	1.921	2.192	2.122	1.523
23 188/15	2.115	2.083	2.044	2.255	2.091	1.926	2.097	2.088	0.882
24 199/A/16	2.158	2.155	2.134	2.197	2.102	1.891	2.158	2.211	1.361
25 167/C/17	2.173	2.158	2.031	2.227	2.235	1.943	2.158	2.210	1.215
26 141/17	2.203	2.126	2.102	2.268	2.237	1.936	2.141	2.178	1.411
27 13/18	2.056	2.151	2.113	**2.301**	2.172	1.953	2.249	2.211	1.484

Strains were incubated at 15°C, 22°C, and 37°C and identified at several time-points (48h, 72h and 7d). The samples were applied on the target plate by the on-target extraction method. Scores presented are the average of up to five measurements. Highlighted fields show probable species level identification scores, while highlighted fields with boldface type log scores show highly probable species level identification.

**Table 2:** Identification results (as log scores) regarding *V. anguillarum* incubation time and temperature for strains cultured on Tryptone soy agar (TSA) containing 0.5% NaCl.

Strain ID	15°C			22°C			37°C		
	48h	72h	7d	48h	72h	7d	48h	72h	7d
1 305/C/02	2.193	2.134	1.928	2.177	2.218	1.747	2.197	2.079	0.814
2 68/05	2.145	2.148	1.988	2.078	2.194	1.744	2.166	2.033	1.319
3 82/05	2.170	2.191	2.056	2.161	2.189	1.818	2.148	1.967	1.558
4 372/05	2.169	2.152	1.965	2.063	2.175	1.794	2.138	2.040	1.527
5 31/06	2.237	2.186	1.972	2.122	2.139	1.893	2.243	1.941	1.260
6 136/C/06	2.241	2.191	1.939	2.195	2.086	1.886	2.159	2.052	1.427
7 77/09	2.202	2.175	1.994	2.107	2.164	1.648	2.126	2.038	1.517
8 135/09	2.263	2.102	2.000	2.074	2.095	1.846	2.164	2.027	0.859
9 158/B/09	2.240	2.173	2.052	2.064	2.197	1.998	2.121	2.068	1.208
10 84/10	2.169	2.184	1.875	2.255	2.265	1.691	2.169	1.985	1.971
11 246/D/10	2.213	2.172	1.977	2.093	2.229	1.867	2.222	2.042	1.610
12 65/A/11	2.160	2.234	2.024	2.107	2.185	1.965	2.274	2.089	2.136
13 83/A/11	2.069	2.137	2.039	2.176	2.198	1.717	2.040	2.293	1.495
14 104/B/12	2.106	2.213	1.980	2.185	2.138	1.892	2.071	2.152	1.397
15 117/13	2.195	2,140	2.045	2.084	2.223	1.767	2.026	2.052	1.535
16 189/13	2.052	2.100	2.023	2.096	2.134	1.871	2.240	2.090	0.927
17 440/14	2.064	2.224	1.922	2.168	2.241	1.803	2.072	1.981	1.306
18 449/A/14	2.004	2.118	1.971	2.107	2.242	1.812	2.096	2.079	1.313
19 4/B/15	2.081	2.217	2.043	2.084	2.222	1.900	2.136	2.118	1.498
20 73/15	2.038	2.089	2.046	2.107	2.110	1.764	2.220	**2.323**	1.365
21 121/15	2.057	2.194	2.008	2.136	2.170	1.694	2.179	2.081	1.281
22 130/F/15	2.133	2.183	2.115	2.194	2.157	1.727	2.173	2.078	1.460
23188/15	2.083	2.084	1.806	2.098	2.121	1.537	2.049	1.947	1.240
24 199/A/16	2.130	2.135	2.032	2.131	2.245	1.795	2.146	2.098	1.454
25 167/C/17	2.222	2.170	1.963	2.072	2.162	1.836	2.079	2.126	1.326
26 141/17	2.281	2.164	2.002	2.166	2.165	1.778	1.827	2.040	1.261
27 13/18	2.206	2.125	2.007	2.164	2.259	1.890	2.169	1.888	1.124

Strains were incubated at 15°C, 22°C, and 37°C and identified at several time-points (48h, 72h and 7d). The samples were applied on the target plate by the on-target extraction method. Scores presented are the average of up to five measurements. Highlighted fields show highly probable species level identification scores, while the highlighted field with boldface type log score shows highly probable species level identification.

The incubation temperature affected the MALDI-TOF MS species identification results significantly (p<0.001), as the 37°C incubation yielded the most unreliable results. Unreliable identifications and no-identifications were growing with the incubation duration at 37°C, on both media, amounting to 88.89% for 7d incubation on supplemented TSA, and 92.60% for 7d incubation on non-supplemented TSA.

The correct identification of *V*. *anguillarum* strains was diminishing with the prolongation of the incubation time, on both media and all temperatures. The 7d incubations enabled correct identifications of less than 30% of overall specimens in various culture conditions, while only for supplemented TSA at 15°C it amounted to 88.89% (Table 3). The logistic regression modelling showed no significant association of strains (p = 0.4) and the final fixed effects model included all variables of culture conditions as independent main effects since no interactions of variables showed a significant improvement of the model. According to K-fold cross validation, the final model had an average accuracy of 93.3% and by the receiver operating characteristics the area under the curve of 0.964. The predicted probabilities for a positive species identification of the final model for all combinations of culture conditions are presented in Table 3.

**Table 3:** Influence of culture conditions (temperature, duration of incubation, culture media (supplementation with 1.5% NaCl)) on the quality of MALDI-TOF mass spectra obtained for 27 strains of *V. anguillarum*.

Culture conditions		ID to genus level only (%)	Observed ID to species level (%) / prediction*	NI (%)
TSA supplemented with 1.5% NaCl	15°C; 48h		100 / 100	
	15°C; 72h	3.70	96.30 / 99.98	
	15°C; 7d	11.11	88.89 / 47.83	
	22°C; 48h		100 / 99.94	
	22°C; 72h		100 / 99.50	
	22°C; 7d	85.19	14.81 / 16.11	
	37°C; 48h		100 / 99.56	
	37°C; 72h		100 / 96.59	
	37°C; 7d	11.11	0 / 2.68	88.89
TSA containing 0.5% NaCl	15°C; 48h		100 / 99.99	
	15°C; 72h		100 / 99.89	
	15°C; 7d	48.15	51.85 / 47.83	
	22°C; 48h		100 / 99.59	
	22°C; 72h		100 / 96.81	
	22°C; 7d	85.19	0 / 2.87	14.81
	37°C; 48	3.70	96.30 / 97.22	
	37°C; 72h	22.22	77.78 / 81.31	
	37°C; 7d	3.70	3.70 / 0.42	92.60

Percentage of identification to genus/species level was calculated for a total number of strains in relation to culture conditions. Log scores <1.700 were considered unreliable and therefore strains with scores <1.700 for particular measurements/conditions were considered as not identifiable (NI).

* Predictions of probabilities for a positive species identification based on a generalized linear logistic regression model where positive identification was taken as a log score > = 2.000.

The NaCl-supplemented TSA enabled the correct identification of *V*. *anguillarum* against the Biotyper database as highly probable species level identification in 2.1% of cases, when observing all culture conditions with that medium. When observing the 22°C incubation at 48h, it amounted to 11.11%.

**Fig 1:**
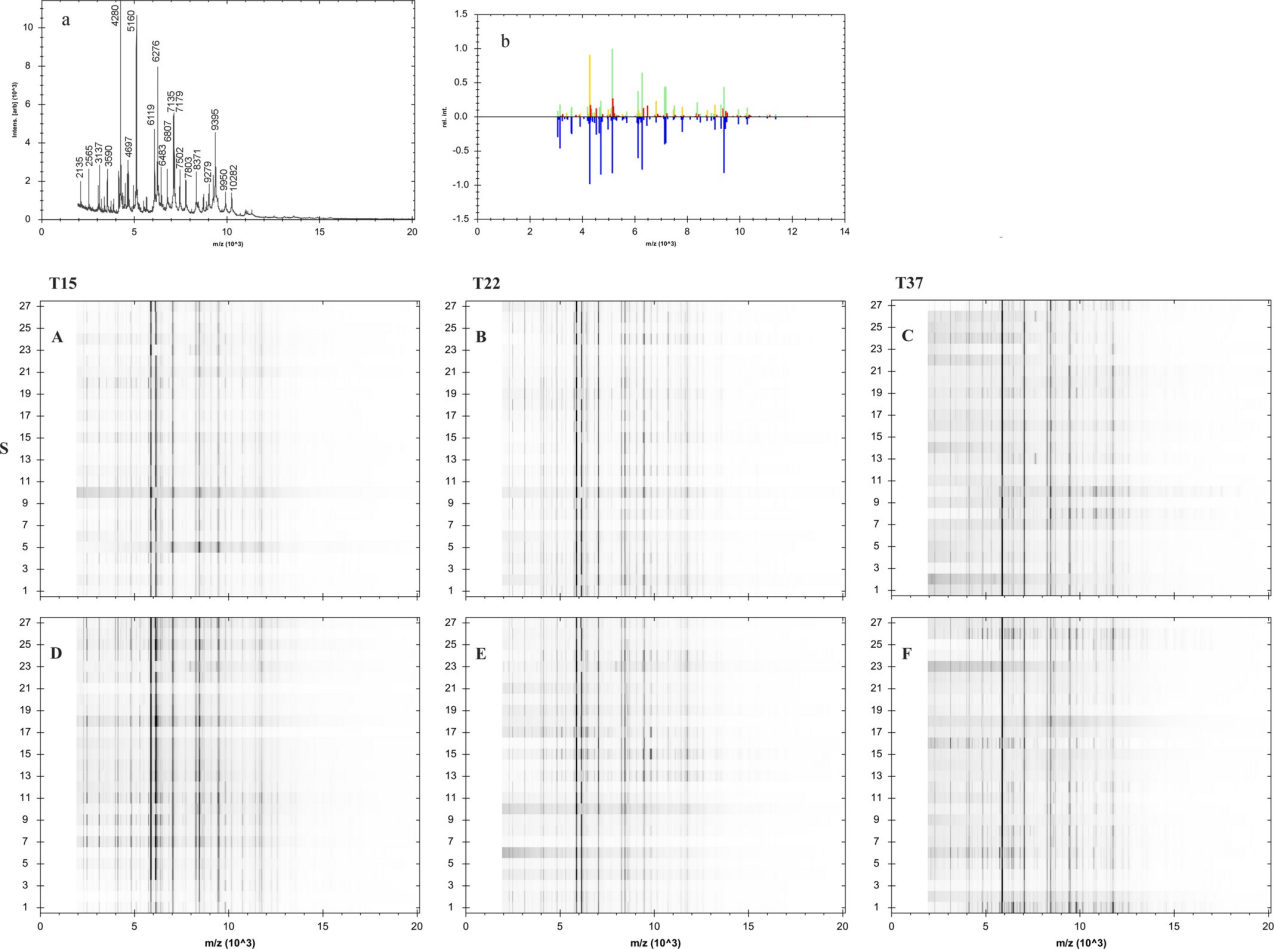
Gelview of signatures obtained from analysis of 27 strains of *Vibrio anguillarum* grown on different media, characterized by mass/charge ratio peak (*m/z*) and frequency of appearance in respect of the supplementation of TSA medium with sodium chloride. Shown is the representative spectrum of *V*. *anguillarum*. The gel view was obtained using MALDI Biotyper 3.0 by displaying a pseudo-gel representation of spectra. Peak intensities were gray-scaled with abscissa values as mass to charge ratios (*m/z*) and spectral numbers as the ordinates.Abbreviations: T15, T22, T37 = temperatures of incubation: 15°C, 22°C, 37°C; S = NaCl-supplemented TSA medium; A-F = strains identified at their first growth on respective medium: A = incubated at 15°C for 48h, B = at 22°C for 48, C = at 37°C for 48h, D = at 15°C for 48h, E = at 22°C for 48h, and F = at 37°C for 48h; a = representative mass spectra, b = mirror view of the best match against the Bruker database.

**Fig 2:**
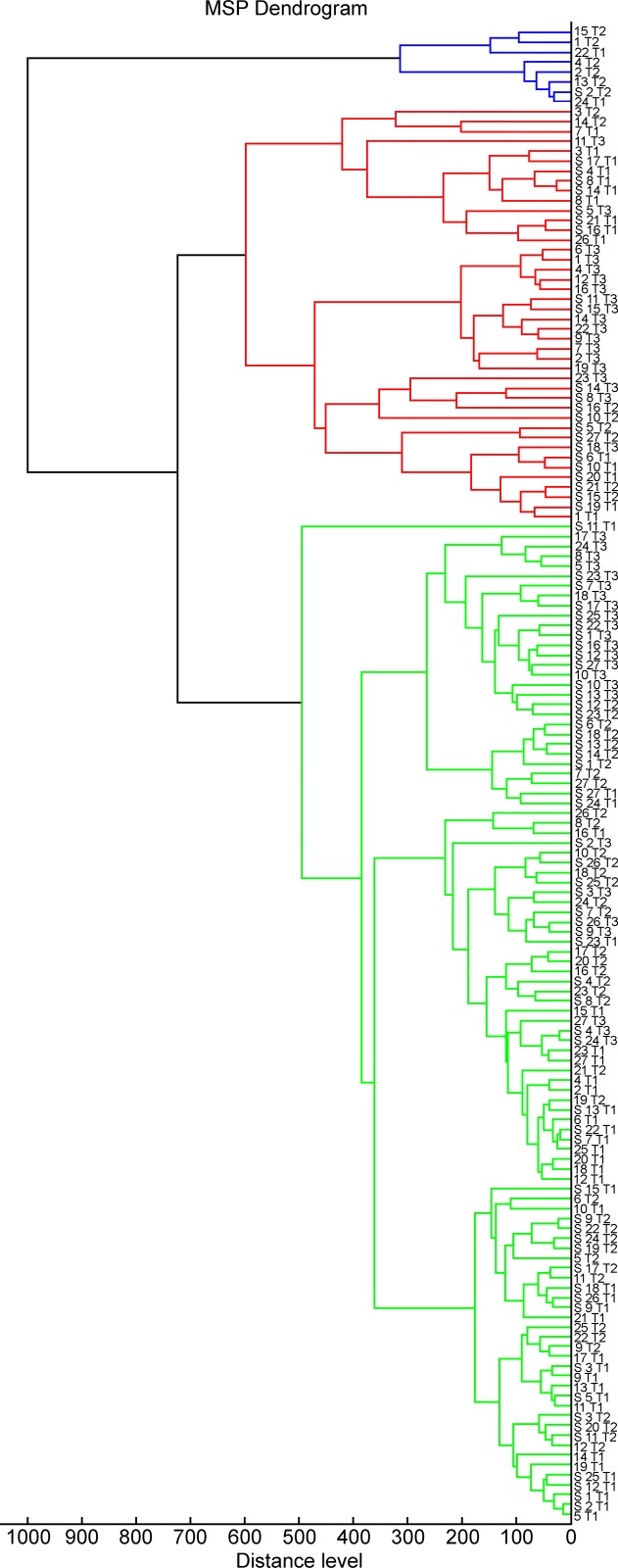
Dendrogram calculated on the basis of cluster analysis of mass spectra of identified *V*. *anguillarum* strains, analysed after three time-points when cultured at 15°C, 22°C, 37°C on TSA supplemented and not supplemented with 1.5% NaCl. Abbreviations: T1 = 15°C, T2 = 22°C, T2 = 37°C; S = NaCl-supplemented TSA medium.

There was a difference between the NaCl supplementation of the culture media and successful acquisition of mass spectra (Figs 1 and 2). The mass spectra of bacteria grown on the supplemented media had the greater number of signals and the higher signal-to-noise ratio. Most of the ions were appearing reproducibly across all the conditions tested, i.e. media, temperature, and incubation duration. The reproducible appearance of these ions at the specific *m/z* ratios were used for comparison of identification results, and the mass spectral quality sufficient for automatic acquisition was on the highest level for specimens grown at 15°C for 72h.

## Discussion

Routine, time-consuming methods for identification of *V*. *anguillarum* could have limited reliability and accuracy. MALDI-TOF MS identification, although rapid and straightforward, needs to be enhanced regarding the choice of the medium type, incubation time and preferred incubation temperature for suspected *V*. *anguillarum* outbreaks, which was the aim of this work, and has not yet been described.

Most of the affected vibriotic fish die without clinical signs. The external clinical signs, when manifested, are not discernible from other fish haemorrhagic septicaemias in their first phases [9], so it is most likely that a general purpose medium for bacterial cultivation would be used, such as tryptone soy agar with or without the addition of sodium chloride. The preferred supplementation of NaCl for *V*. *anguillarum* varies between 0.5–3% (w/v) [5]. The strain growth can be influenced by the salinity of the medium, but no significant differences were found between 0.5 and 1.5% NaCl, while 3% reduced the growth rate [12]. In our work, MALDI-TOF MS identification score was significantly higher in the 1.5% NaCl-supplemented medium than in the non-supplemented one.

Temperature, however, serves as a more detrimental factor for *V*. *anguillarum* growth than salinity of the media [1], which we also noticed in our strains. A number of no-growths was recorded on both media when cultivated at 37°C, although the percentage of strains not growing at that temperature was higher for non-supplemented medium. It is recommended that a suspected *V*. *anguillarum* be incubated at 15–25°C up to 7 days [5]. However, as its growth rate is known to increase with temperature, Hickey and Lee [1] state that it grows best between 30 and 34°C, with a maximum growth temperature of 38.5°C. Our strains did not all grow at 37°C in all culture conditions, particularly on the non-enriched medium at the 72h time-point (4.94%). Indeed, low osmolarity, through an osmotic shock, induced morphological modifications in *V*. *anguillarum* cultured at 37°C in the work of Piccininno et al. [13]. MALDI-TOF MS had a lower identification score on our isolates grown at higher temperatures, particularly on those incubated for 7 days. Other two incubation temperatures, when only cultures of 48h and 72h are considered, yielded high identification scores, especially for the NaCl-enriched TSA.

Thus, the age of the cultured strains and use of media did impact the mass spectra. The quality of the spectra was correctly expected to reduce with the age of the strains, as cells in the log phase of growth would be ideal for identification since MALDI-TOF MS recognizes mostly 16S ribosomal proteins [14]. Also, we expected that by virtue of dominance of ribosomal proteins’ peaks with house-keeping functions they would stay unchanged under different growth conditions [15], albeit different media change metabolic needs of cultivated bacteria [16]. The vast majority of isolates were correctly identified under all culture conditions (15.22% to the genus level only and 73.85% to the species level), but only a fraction had a score value ≥ 2.300, which is a threshold for highly probable species level identification. However, for most (aquatic) bacteria, a less restrictive cut-off score (≥ 2.000) is tolerated for a secure identification [17]. Such a score was retrieved for *V*. *anguillarum* strains grown on NaCl-supplemented TSA at 22°C and incubated for 48h (100% identification to the species level), as the highest overall score of this work (2.232).

All seven discriminating peaks of *V*. *anguillarum* were found in all isolates identified, confirming their uniformness for rapid identification. Identification quality is correlated with the quality of the acquired spectrum, and its inclusion in the database. The peaks were measured out of the spectral replicates in reference signatures, and the frequency of appearance or reproducibility gave significance to each peak obtained [16]. The strain differentiation is based on a limited number of peaks, therefore the quality of mass spectra could influence strain differentiation [18].

Fish affected by vibriosis caused by *V*. *anguillarum* show a number of, often non-specific, external clinical signs, including lethargy, red spots on ventral and lateral sides of body, and ulcerative lesions. In affected fish *V*. *anguillarum* can also be found in high concentrations in the blood and abdominal fluid, which accumulates as a clear, viscous liquid [9]. Since MALDI-TOF MS can identify pathogens from blood and other bodily fluids rapidly and accurately, in order to save culturing time, procedures were developed for identification of bacteria directly from human clinical samples [19–21]. Such an approach of using MALDI-TOF MS on fish body fluids would greatly benefit accurate diagnosis and treatment, which could influence mortality of fish affected by vibriosis and shorten the turnaround time of *V*. *anguillarum* identification. Although MALDI-TOF MS is a highly reproducible method for rapid discrimination of *Vibrio* species, its database should be extended with more of the aquatic species, and *V*. *anguillarum* in particular. Until the further development of the direct method, suspected marine *V*. *anguillarum* should be grown on a NaCl-supplemented medium at temperatures between 15 and 22°C. MALDI-TOF MS protein fingerprinting with the on-target extraction should be performed upon the occurrence of first colonies up to 72h. Due to its high throughput and differentiation capacity of closely related Vibrio strains, MALDI-TOF MS should become an integrated tool of microbiological laboratories dealing with aquatic pathogens.

## Supporting information

S1 Table Raw data. (XLSX)
